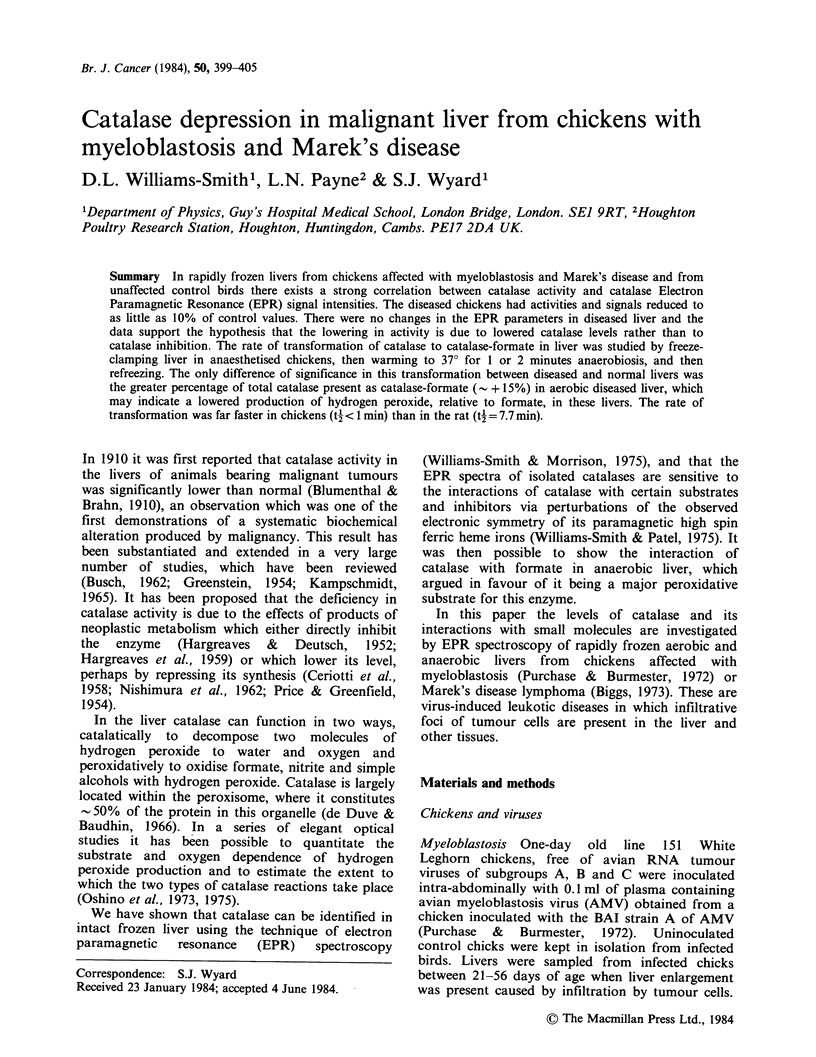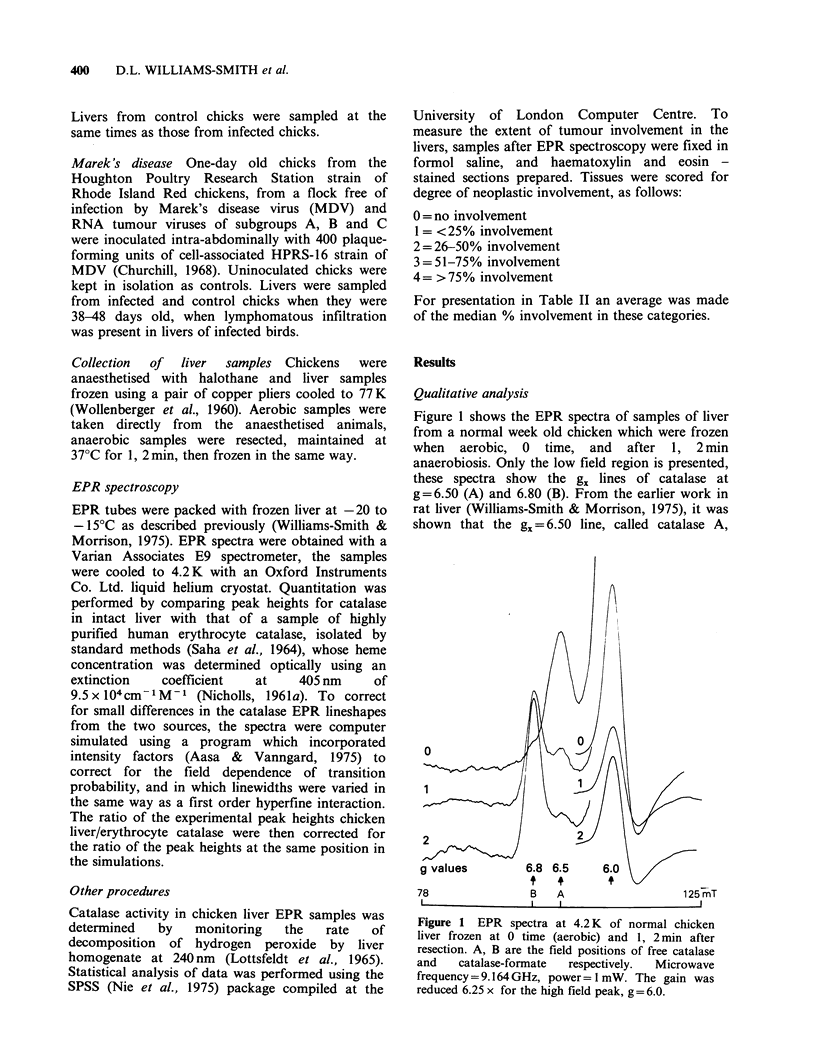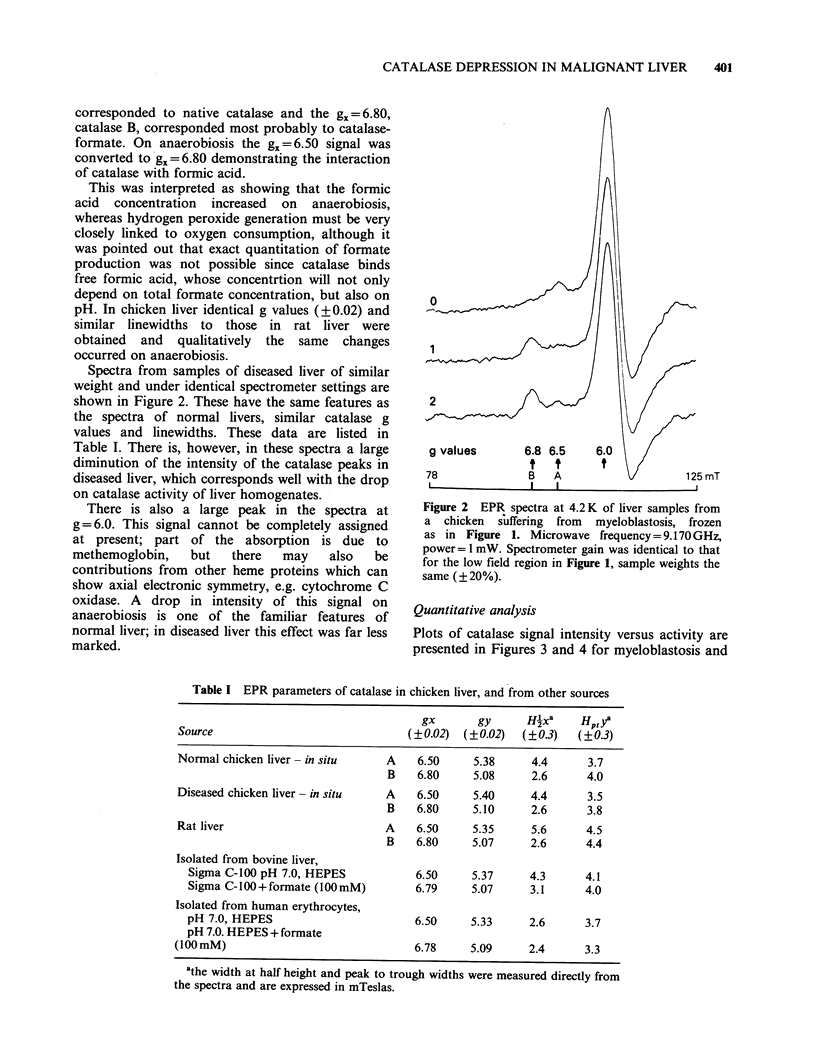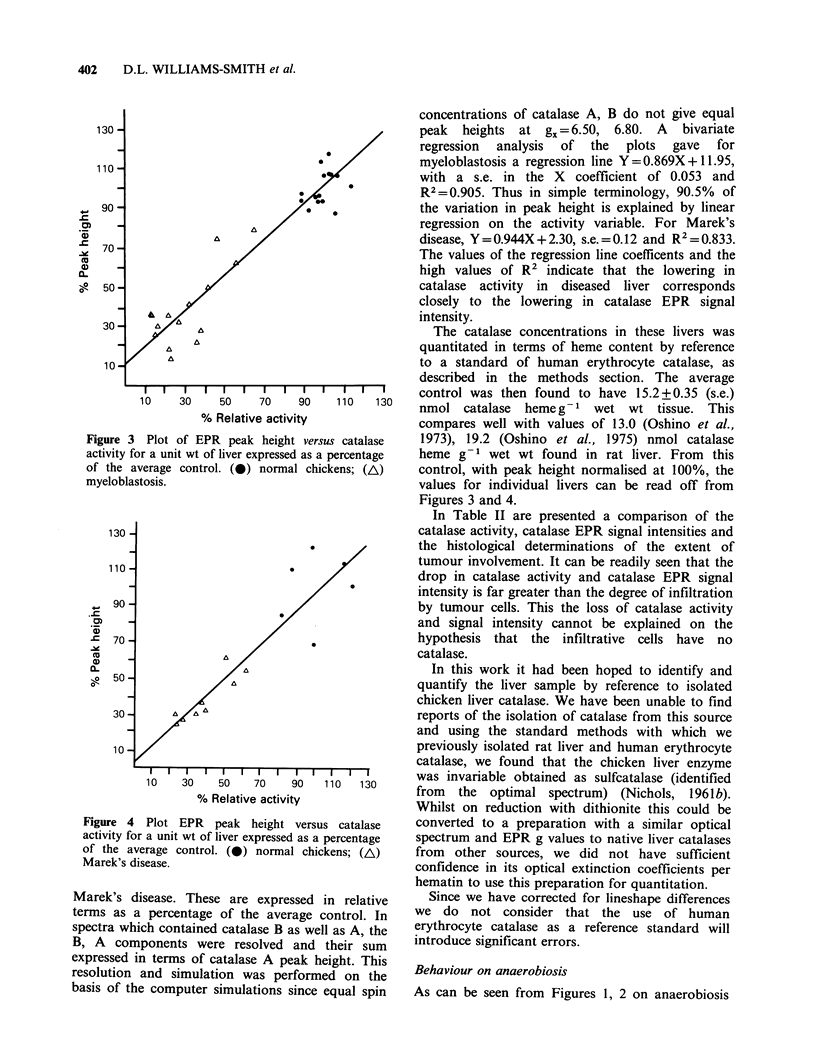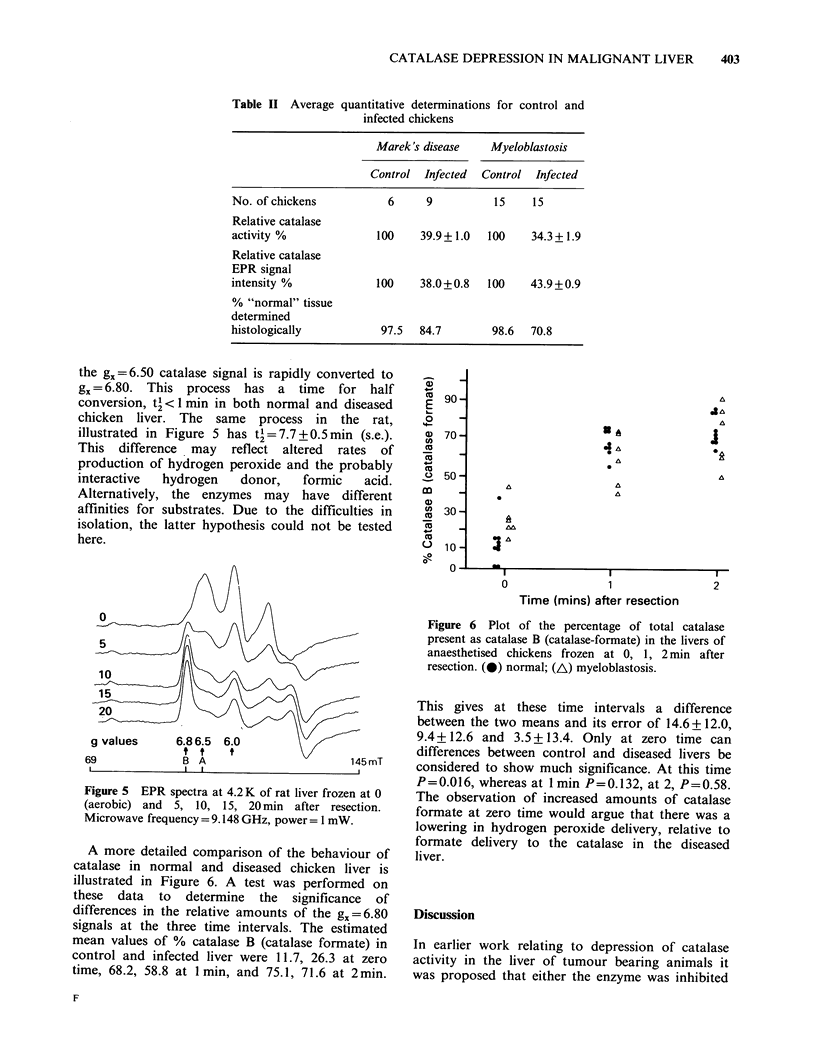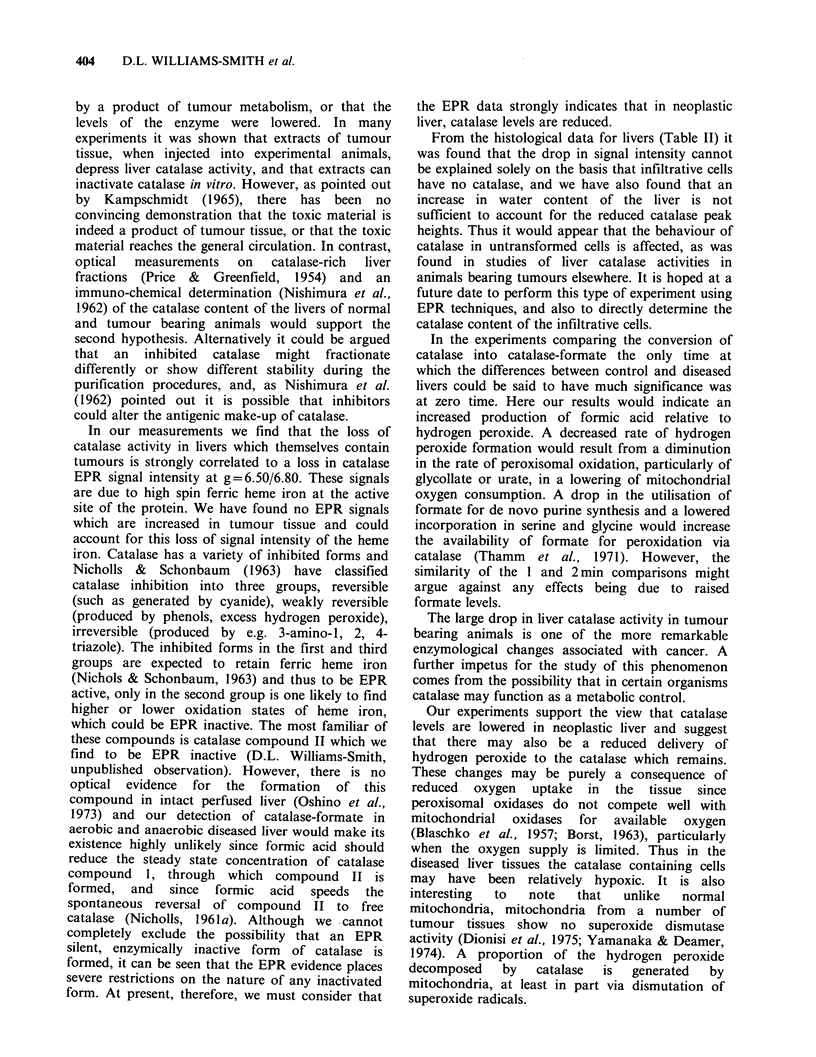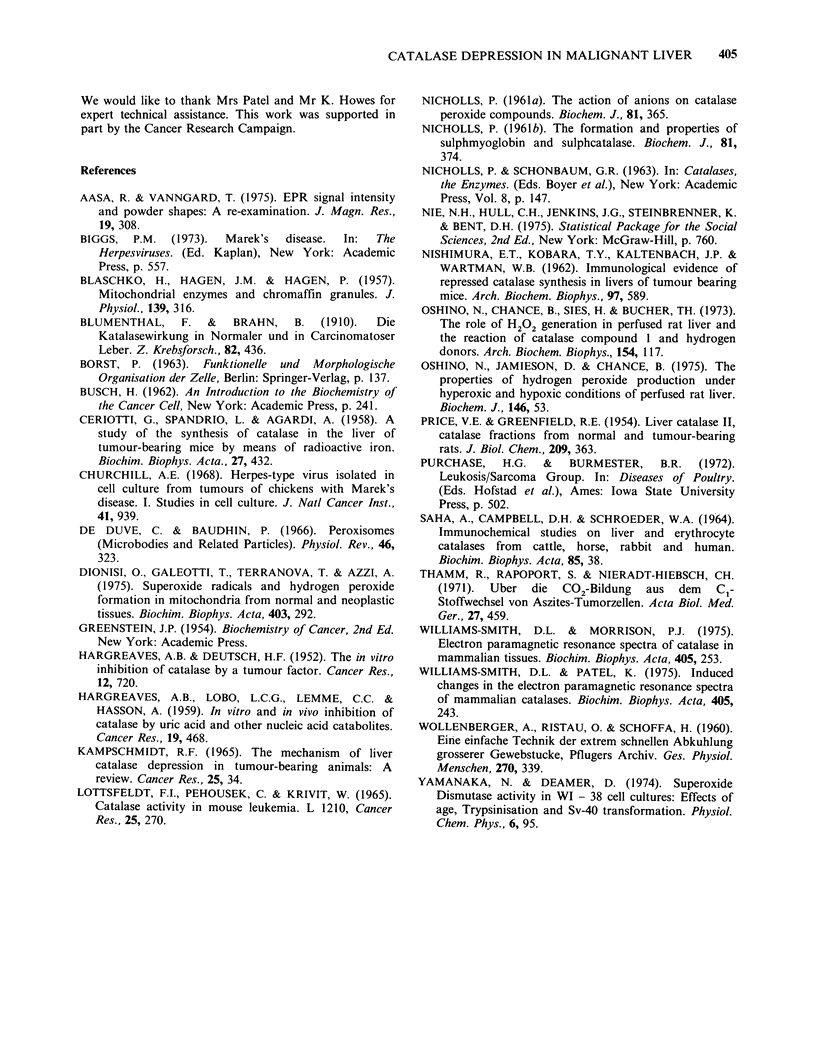# Catalase depression in malignant liver from chickens with myeloblastosis and Marek's disease.

**DOI:** 10.1038/bjc.1984.189

**Published:** 1984-09

**Authors:** D. L. Williams-Smith, L. N. Payne, S. J. Wyard

## Abstract

In rapidly frozen livers from chickens affected with myeloblastosis and Marek's disease and from unaffected control birds there exists a strong correlation between catalase activity and catalase Electron Paramagnetic Resonance (EPR) signal intensities. The diseased chickens had activities and signals reduced to as little as 10% of control values. There were no changes in the EPR parameters in diseased liver and the data support the hypothesis that the lowering in activity is due to lowered catalase levels rather than to catalase inhibition. The rate of transformation of catalase to catalase-formate in liver was studied by freeze-clamping liver in anaesthetised chickens, then warming to 37 degrees for 1 or 2 minutes anaerobiosis, and then refreezing. The only difference of significance in this transformation between diseased and normal livers was the greater percentage of total catalase present as catalase-formate (approximately + 15%) in aerobic diseased liver, which may indicate a lowered production of hydrogen peroxide, relative to formate, in these livers. The rate of transformation was far faster in chickens (t1/2 less than 1 min) than in the rat (t1/2 = 7.7 min).


*Br. J. Cancer* (1984), **50**, 399–405

### Catalase depression in malignant liver from chickens with myeloblastosis and Marek's disease

D.L. Williams-Smith[1], L.N. Payne[2] & S.J. Wyard[1]

[1]*Department of Physics, Guy's Hospital Medical School, London Bridge, London. SE1 9RT,* [2]*Houghton Poultry Research Station, Houghton, Huntingdon, Cambs. PE17 2DA UK.*

**Summary** In rapidly frozen livers from chickens affected with myeloblastosis and Marek's disease and from unaffected control birds there exists a strong correlation between catalase activity and catalase Electron Paramagnetic Resonance (EPR) signal intensities. The diseased chickens had activities and signals reduced to as little as 10% of control values. There were no changes in the EPR parameters in diseased liver and the data support the hypothesis that the lowering in activity is due to lowered catalase levels rather than to catalase inhibition. The rate of transformation of catalase to catalase-formate in liver was studied by freeze-clamping liver in anaesthetised chickens, then warming to 37° for 1 or 2 minutes anaerobiosis, and then refreezing. The only difference of significance in this transformation between diseased and normal livers was the greater percentage of total catalase present as catalase-formate ($\sim +15\%$) in aerobic diseased liver, which may indicate a lowered production of hydrogen peroxide, relative to formate, in these livers. The rate of transformation was far faster in chickens ($t\frac{1}{2} < 1$ min) than in the rat ($t\frac{1}{2} = 7.7$ min).

In 1910 it was first reported that catalase activity in the livers of animals bearing malignant tumours was significantly lower than normal (Blumenthal & Brahn, 1910), an observation which was one of the first demonstrations of a systematic biochemical alteration produced by malignancy. This result has been substantiated and extended in a very large number of studies, which have been reviewed (Busch, 1962; Greenstein, 1954; Kampschmidt, 1965). It has been proposed that the deficiency in catalase activity is due to the effects of products of neoplastic metabolism which either directly inhibit the enzyme (Hargreaves & Deutsch, 1952; Hargreaves *et al.*, 1959) or which lower its level, perhaps by repressing its synthesis (Ceriotti *et al.*, 1958; Nishimura *et al.*, 1962; Price & Greenfield, 1954).

In the liver catalase can function in two ways, catalatically to decompose two molecules of hydrogen peroxide to water and oxygen and peroxidatively to oxidise formate, nitrite and simple alcohols with hydrogen peroxide. Catalase is largely located within the peroxisome, where it constitutes ~50% of the protein in this organelle (de Duve & Baudhin, 1966). In a series of elegant optical studies it has been possible to quantitate the substrate and oxygen dependence of hydrogen peroxide production and to estimate the extent to which the two types of catalase reactions take place (Oshino *et al.*, 1973, 1975).

We have shown that catalase can be identified in intact frozen liver using the technique of electron paramagnetic resonance (EPR) spectroscopy (Williams-Smith & Morrison, 1975), and that the EPR spectra of isolated catalases are sensitive to the interactions of catalase with certain substrates and inhibitors via perturbations of the observed electronic symmetry of its paramagnetic high spin ferric heme irons (Williams-Smith & Patel, 1975). It was then possible to show the interaction of catalase with formate in anaerobic liver, which argued in favour of it being a major peroxidative substrate for this enzyme.

In this paper the levels of catalase and its interactions with small molecules are investigated by EPR spectroscopy of rapidly frozen aerobic and anaerobic livers from chickens affected with myeloblastosis (Purchase & Burmester, 1972) or Marek's disease lymphoma (Biggs, 1973). These are virus-induced leukotic diseases in which infiltrative foci of tumour cells are present in the liver and other tissues.

### Materials and methods

### *Chickens and viruses*

*Myeloblastosis* One-day old line 151 White Leghorn chickens, free of avian RNA tumour viruses of subgroups A, B and C were inoculated intra-abdominally with 0.1 ml of plasma containing avian myeloblastosis virus (AMV) obtained from a chicken inoculated with the BAI strain A of AMV (Purchase & Burmester, 1972). Uninoculated control chicks were kept in isolation from infected birds. Livers were sampled from infected chicks between 21–56 days of age when liver enlargement was present caused by infiltration by tumour cells.

Correspondence: S.J. Wyard
Received 23 January 1984; accepted 4 June 1984.

© The Macmillan Press Ltd., 1984

Livers from control chicks were sampled at the same times as those from infected chicks.

*Marek's disease* One-day old chicks from the Houghton Poultry Research Station strain of Rhode Island Red chickens, from a flock free of infection by Marek's disease virus (MDV) and RNA tumour viruses of subgroups A, B and C were inoculated intra-abdominally with 400 plaque-forming units of cell-associated HPRS-16 strain of MDV (Churchill, 1968). Uninoculated chicks were kept in isolation as controls. Livers were sampled from infected and control chicks when they were 38–48 days old, when lymphomatous infiltration was present in livers of infected birds.

*Collection of liver samples* Chickens were anaesthetised with halothane and liver samples frozen using a pair of copper pliers cooled to 77 K (Wollenberger *et al.*, 1960). Aerobic samples were taken directly from the anaesthetised animals, anaerobic samples were resected, maintained at 37°C for 1, 2 min, then frozen in the same way.

*EPR spectroscopy*

EPR tubes were packed with frozen liver at −20 to −15°C as described previously (Williams-Smith & Morrison, 1975). EPR spectra were obtained with a Varian Associates E9 spectrometer, the samples were cooled to 4.2 K with an Oxford Instruments Co. Ltd. liquid helium cryostat. Quantitation was performed by comparing peak heights for catalase in intact liver with that of a sample of highly purified human erythrocyte catalase, isolated by standard methods (Saha *et al.*, 1964), whose heme concentration was determined optically using an extinction coefficient at 405 nm of $9.5 \times 10^4 cm^{-1} M^{-1}$ (Nicholls, 1961*a*). To correct for small differences in the catalase EPR lineshapes from the two sources, the spectra were computer simulated using a program which incorporated intensity factors (Aasa & Vanngard, 1975) to correct for the field dependence of transition probability, and in which linewidths were varied in the same way as a first order hyperfine interaction. The ratio of the experimental peak heights chicken liver/erythrocyte catalase were then corrected for the ratio of the peak heights at the same position in the simulations.

*Other procedures*

Catalase activity in chicken liver EPR samples was determined by monitoring the rate of decomposition of hydrogen peroxide by liver homogenate at 240 nm (Lottsfeldt *et al.*, 1965). Statistical analysis of data was performed using the SPSS (Nie *et al.*, 1975) package compiled at the University of London Computer Centre. To measure the extent of tumour involvement in the livers, samples after EPR spectroscopy were fixed in formol saline, and haematoxylin and eosin – stained sections prepared. Tissues were scored for degree of neoplastic involvement, as follows:

0 = no involvement
1 = <25% involvement
2 = 26–50% involvement
3 = 51–75% involvement
4 = >75% involvement

For presentation in Table II an average was made of the median % involvement in these categories.

### Results

*Qualitative analysis*

Figure 1 shows the EPR spectra of samples of liver from a normal week old chicken which were frozen when aerobic, 0 time, and after 1, 2 min anaerobiosis. Only the low field region is presented, these spectra show the $g_x$ lines of catalase at $g = 6.50$ (A) and 6.80 (B). From the earlier work in rat liver (Williams-Smith & Morrison, 1975), it was shown that the $g_x = 6.50$ line, called catalase A,

**Figure 1** EPR spectra at 4.2 K of normal chicken liver frozen at 0 time (aerobic) and 1, 2 min after resection. A, B are the field positions of free catalase and catalase-formate respectively. Microwave frequency = 9.164 GHz, power = 1 mW. The gain was reduced 6.25× for the high field peak, g = 6.0.

corresponded to native catalase and the $g_x = 6.80$, catalase B, corresponded most probably to catalase-formate. On anaerobiosis the $g_x = 6.50$ signal was converted to $g_x = 6.80$ demonstrating the interaction of catalase with formic acid.

This was interpreted as showing that the formic acid concentration increased on anaerobiosis, whereas hydrogen peroxide generation must be very closely linked to oxygen consumption, although it was pointed out that exact quantitation of formate production was not possible since catalase binds free formic acid, whose concentrtion will not only depend on total formate concentration, but also on pH. In chicken liver identical g values ($\pm 0.02$) and similar linewidths to those in rat liver were obtained and qualitatively the same changes occurred on anaerobiosis.

Spectra from samples of diseased liver of similar weight and under identical spectrometer settings are shown in Figure 2. These have the same features as the spectra of normal livers, similar catalase g values and linewidths. These data are listed in Table I. There is, however, in these spectra a large diminution of the intensity of the catalase peaks in diseased liver, which corresponds well with the drop on catalase activity of liver homogenates.

There is also a large peak in the spectra at $g = 6.0$. This signal cannot be completely assigned at present; part of the absorption is due to methemoglobin, but there may also be contributions from other heme proteins which can show axial electronic symmetry, e.g. cytochrome C oxidase. A drop in intensity of this signal on anaerobiosis is one of the familiar features of normal liver; in diseased liver this effect was far less marked.

**Figure 2** EPR spectra at 4.2 K of liver samples from a chicken suffering from myeloblastosis, frozen as in **Figure 1.** Microwave frequency = 9.170 GHz, power = 1 mW. Spectrometer gain was identical to that for the low field region in **Figure 1**, sample weights the same ($\pm 20\%$).

### *Quantitative analysis*

Plots of catalase signal intensity versus activity are presented in Figures 3 and 4 for myeloblastosis and

**Table I** EPR parameters of catalase in chicken liver, and from other sources

| *Source* | | *gx* (±*0.02*) | *gy* (±*0.02*) | $H\frac{1}{2}x^a$ (±*0.3*) | $H_{pt}y^a$ (±*0.3*) |
|---|---|---|---|---|---|
| Normal chicken liver – *in situ* | A | 6.50 | 5.38 | 4.4 | 3.7 |
| | B | 6.80 | 5.08 | 2.6 | 4.0 |
| Diseased chicken liver – *in situ* | A | 6.50 | 5.40 | 4.4 | 3.5 |
| | B | 6.80 | 5.10 | 2.6 | 3.8 |
| Rat liver | A | 6.50 | 5.35 | 5.6 | 4.5 |
| | B | 6.80 | 5.07 | 2.6 | 4.4 |
| Isolated from bovine liver, | | | | | |
| Sigma C-100 pH 7.0, HEPES | | 6.50 | 5.37 | 4.3 | 4.1 |
| Sigma C-100 + formate (100 mM) | | 6.79 | 5.07 | 3.1 | 4.0 |
| Isolated from human erythrocytes, | | | | | |
| pH 7.0, HEPES | | 6.50 | 5.33 | 2.6 | 3.7 |
| pH 7.0. HEPES + formate (100 mM) | | 6.78 | 5.09 | 2.4 | 3.3 |

[a]the width at half height and peak to trough widths were measured directly from the spectra and are expressed in mTeslas.

**Figure 3** Plot of EPR peak height *versus* catalase activity for a unit wt of liver expressed as a percentage of the average control. (●) normal chickens; (△) myeloblastosis.

**Figure 4** Plot EPR peak height versus catalase activity for a unit wt of liver expressed as a percentage of the average control. (●) normal chickens; (△) Marek's disease.

Marek's disease. These are expressed in relative terms as a percentage of the average control. In spectra which contained catalase B as well as A, the B, A components were resolved and their sum expressed in terms of catalase A peak height. This resolution and simulation was performed on the basis of the computer simulations since equal spin concentrations of catalase A, B do not give equal peak heights at $g_x = 6.50$, 6.80. A bivariate regression analysis of the plots gave for myeloblastosis a regression line $Y = 0.869X + 11.95$, with a s.e. in the X coefficient of 0.053 and $R^2 = 0.905$. Thus in simple terminology, 90.5% of the variation in peak height is explained by linear regression on the activity variable. For Marek's disease, $Y = 0.944X + 2.30$, s.e. $= 0.12$ and $R^2 = 0.833$. The values of the regression line coefficents and the high values of $R^2$ indicate that the lowering in catalase activity in diseased liver corresponds closely to the lowering in catalase EPR signal intensity.

The catalase concentrations in these livers was quantitated in terms of heme content by reference to a standard of human erythrocyte catalase, as described in the methods section. The average control was then found to have $15.2 \pm 0.35$ (s.e.) nmol catalase heme $g^{-1}$ wet wt tissue. This compares well with values of 13.0 (Oshino *et al.*, 1973), 19.2 (Oshino *et al.*, 1975) nmol catalase heme $g^{-1}$ wet wt found in rat liver. From this control, with peak height normalised at 100%, the values for individual livers can be read off from Figures 3 and 4.

In Table II are presented a comparison of the catalase activity, catalase EPR signal intensities and the histological determinations of the extent of tumour involvement. It can be readily seen that the drop in catalase activity and catalase EPR signal intensity is far greater than the degree of infiltration by tumour cells. This the loss of catalase activity and signal intensity cannot be explained on the hypothesis that the infiltrative cells have no catalase.

In this work it had been hoped to identify and quantify the liver sample by reference to isolated chicken liver catalase. We have been unable to find reports of the isolation of catalase from this source and using the standard methods with which we previously isolated rat liver and human erythrocyte catalase, we found that the chicken liver enzyme was invariable obtained as sulfcatalase (identified from the optimal spectrum) (Nichols, 1961*b*). Whilst on reduction with dithionite this could be converted to a preparation with a similar optical spectrum and EPR g values to native liver catalases from other sources, we did not have sufficient confidence in its optical extinction coefficients per hematin to use this preparation for quantitation.

Since we have corrected for lineshape differences we do not consider that the use of human erythrocyte catalase as a reference standard will introduce significant errors.

*Behaviour on anaerobiosis*

As can be seen from Figures 1, 2 on anaerobiosis

**Table II** Average quantitative determinations for control and infected chickens

| | *Marek's disease* | | *Myeloblastosis* | |
|---|---|---|---|---|
| | *Control* | *Infected* | *Control* | *Infected* |
| No. of chickens | 6 | 9 | 15 | 15 |
| Relative catalase activity % | 100 | 39.9±1.0 | 100 | 34.3±1.9 |
| Relative catalase EPR signal intensity % | 100 | 38.0±0.8 | 100 | 43.9±0.9 |
| % "normal" tissue determined histologically | 97.5 | 84.7 | 98.6 | 70.8 |

the $g_x=6.50$ catalase signal is rapidly converted to $g_x=6.80$. This process has a time for half conversion, $t_{\frac{1}{2}}<1$ min in both normal and diseased chicken liver. The same process in the rat, illustrated in Figure 5 has $t_{\frac{1}{2}}=7.7\pm0.5$ min (s.e.). This difference may reflect altered rates of production of hydrogen peroxide and the probably interactive hydrogen donor, formic acid. Alternatively, the enzymes may have different affinities for substrates. Due to the difficulties in isolation, the latter hypothesis could not be tested here.

**Figure 5** EPR spectra at 4.2 K of rat liver frozen at 0 (aerobic) and 5, 10, 15, 20 min after resection. Microwave frequency = 9.148 GHz, power = 1 mW.

A more detailed comparison of the behaviour of catalase in normal and diseased chicken liver is illustrated in Figure 6. A test was performed on these data to determine the significance of differences in the relative amounts of the $g_x=6.80$ signals at the three time intervals. The estimated mean values of % catalase B (catalase formate) in control and infected liver were 11.7, 26.3 at zero time, 68.2, 58.8 at 1 min, and 75.1, 71.6 at 2 min.

**Figure 6** Plot of the percentage of total catalase present as catalase B (catalase-formate) in the livers of anaesthetised chickens frozen at 0, 1, 2 min after resection. (●) normal; (△) myeloblastosis.

This gives at these time intervals a difference between the two means and its error of 14.6±12.0, 9.4±12.6 and 3.5±13.4. Only at zero time can differences between control and diseased livers be considered to show much significance. At this time $P=0.016$, whereas at 1 min $P=0.132$, at 2, $P=0.58$. The observation of increased amounts of catalase formate at zero time would argue that there was a lowering in hydrogen peroxide delivery, relative to formate delivery to the catalase in the diseased liver.

### Discussion

In earlier work relating to depression of catalase activity in the liver of tumour bearing animals it was proposed that either the enzyme was inhibited by a product of tumour metabolism, or that the levels of the enzyme were lowered. In many experiments it was shown that extracts of tumour tissue, when injected into experimental animals, depress liver catalase activity, and that extracts can inactivate catalase *in vitro*. However, as pointed out by Kampschmidt (1965), there has been no convincing demonstration that the toxic material is indeed a product of tumour tissue, or that the toxic material reaches the general circulation. In contrast, optical measurements on catalase-rich liver fractions (Price & Greenfield, 1954) and an immuno-chemical determination (Nishimura *et al.*, 1962) of the catalase content of the livers of normal and tumour bearing animals would support the second hypothesis. Alternatively it could be argued that an inhibited catalase might fractionate differently or show different stability during the purification procedures, and, as Nishimura *et al.* (1962) pointed out it is possible that inhibitors could alter the antigenic make-up of catalase.

In our measurements we find that the loss of catalase activity in livers which themselves contain tumours is strongly correlated to a loss in catalase EPR signal intensity at $g = 6.50/6.80$. These signals are due to high spin ferric heme iron at the active site of the protein. We have found no EPR signals which are increased in tumour tissue and could account for this loss of signal intensity of the heme iron. Catalase has a variety of inhibited forms and Nicholls & Schonbaum (1963) have classified catalase inhibition into three groups, reversible (such as generated by cyanide), weakly reversible (produced by phenols, excess hydrogen peroxide), irreversible (produced by e.g. 3-amino-1, 2, 4-triazole). The inhibited forms in the first and third groups are expected to retain ferric heme iron (Nichols & Schonbaum, 1963) and thus to be EPR active, only in the second group is one likely to find higher or lower oxidation states of heme iron, which could be EPR inactive. The most familiar of these compounds is catalase compound II which we find to be EPR inactive (D.L. Williams-Smith, unpublished observation). However, there is no optical evidence for the formation of this compound in intact perfused liver (Oshino *et al.*, 1973) and our detection of catalase-formate in aerobic and anaerobic diseased liver would make its existence highly unlikely since formic acid should reduce the steady state concentration of catalase compound 1, through which compound II is formed, and since formic acid speeds the spontaneous reversal of compound II to free catalase (Nicholls, 1961*a*). Although we cannot completely exclude the possibility that an EPR silent, enzymically inactive form of catalase is formed, it can be seen that the EPR evidence places severe restrictions on the nature of any inactivated form. At present, therefore, we must consider that the EPR data strongly indicates that in neoplastic liver, catalase levels are reduced.

From the histological data for livers (Table II) it was found that the drop in signal intensity cannot be explained solely on the basis that infiltrative cells have no catalase, and we have also found that an increase in water content of the liver is not sufficient to account for the reduced catalase peak heights. Thus it would appear that the behaviour of catalase in untransformed cells is affected, as was found in studies of liver catalase activities in animals bearing tumours elsewhere. It is hoped at a future date to perform this type of experiment using EPR techniques, and also to directly determine the catalase content of the infiltrative cells.

In the experiments comparing the conversion of catalase into catalase-formate the only time at which the differences between control and diseased livers could be said to have much significance was at zero time. Here our results would indicate an increased production of formic acid relative to hydrogen peroxide. A decreased rate of hydrogen peroxide formation would result from a diminution in the rate of peroxisomal oxidation, particularly of glycollate or urate, in a lowering of mitochondrial oxygen consumption. A drop in the utilisation of formate for de novo purine synthesis and a lowered incorporation in serine and glycine would increase the availability of formate for peroxidation via catalase (Thamm *et al.*, 1971). However, the similarity of the 1 and 2 min comparisons might argue against any effects being due to raised formate levels.

The large drop in liver catalase activity in tumour bearing animals is one of the more remarkable enzymological changes associated with cancer. A further impetus for the study of this phenomenon comes from the possibility that in certain organisms catalase may function as a metabolic control.

Our experiments support the view that catalase levels are lowered in neoplastic liver and suggest that there may also be a reduced delivery of hydrogen peroxide to the catalase which remains. These changes may be purely a consequence of reduced oxygen uptake in the tissue since peroxisomal oxidases do not compete well with mitochondrial oxidases for available oxygen (Blaschko *et al.*, 1957; Borst, 1963), particularly when the oxygen supply is limited. Thus in the diseased liver tissues the catalase containing cells may have been relatively hypoxic. It is also interesting to note that unlike normal mitochondria, mitochondria from a number of tumour tissues show no superoxide dismutase activity (Dionisi *et al.*, 1975; Yamanaka & Deamer, 1974). A proportion of the hydrogen peroxide decomposed by catalase is generated by mitochondria, at least in part via dismutation of superoxide radicals.

We would like to thank Mrs Patel and Mr K. Howes for expert technical assistance. This work was supported in part by the Cancer Research Campaign.

**References**

AASA, R. & VANNGARD, T. (1975). EPR signal intensity and powder shapes: A re-examination. *J. Magn. Res.*, **19**, 308.

BIGGS, P.M. (1973). Marek's disease. In: *The Herpesviruses*. (Ed. Kaplan), New York: Academic Press, p. 557.

BLASCHKO, H., HAGEN, J.M. & HAGEN, P. (1957). Mitochondrial enzymes and chromaffin granules. *J. Physiol.*, **139**, 316.

BLUMENTHAL, F. & BRAHN, B. (1910). Die Katalasewirkung in Normaler und in Carcinomatoser Leber. *Z. Krebsforsch.*, **82**, 436.

BORST, P. (1963). *Funktionelle und Morphologische Organisation der Zelle*, Berlin: Springer-Verlag, p. 137.

BUSCH, H. (1962). *An Introduction to the Biochemistry of the Cancer Cell*, New York: Academic Press, p. 241.

CERIOTTI, G., SPANDRIO, L. & AGARDI, A. (1958). A study of the synthesis of catalase in the liver of tumour-bearing mice by means of radioactive iron. *Biochim. Biophys. Acta.*, **27**, 432.

CHURCHILL, A.E. (1968). Herpes-type virus isolated in cell culture from tumours of chickens with Marek's disease. I. Studies in cell culture. *J. Natl Cancer Inst.*, **41**, 939.

DE DUVE, C. & BAUDHIN, P. (1966). Peroxisomes (Microbodies and Related Particles). *Physiol. Rev.*, **46**, 323.

DIONISI, O., GALEOTTI, T., TERRANOVA, T. & AZZI, A. (1975). Superoxide radicals and hydrogen peroxide formation in mitochondria from normal and neoplastic tissues. *Biochim. Biophys. Acta*, **403**, 292.

GREENSTEIN, J.P. (1954). *Biochemistry of Cancer, 2nd Ed.* New York: Academic Press.

HARGREAVES, A.B. & DEUTSCH, H.F. (1952). The *in vitro* inhibition of catalase by a tumour factor. *Cancer Res.*, **12**, 720.

HARGREAVES, A.B., LOBO, L.C.G., LEMME, C.C. & HASSON, A. (1959). *In vitro* and *in vivo* inhibition of catalase by uric acid and other nucleic acid catabolites. *Cancer Res.*, **19**, 468.

KAMPSCHMIDT, R.F. (1965). The mechanism of liver catalase depression in tumour-bearing animals: A review. *Cancer Res.*, **25**, 34.

LOTTSFELDT, F.I., PEHOUSEK, C. & KRIVIT, W. (1965). Catalase activity in mouse leukemia. L 1210, *Cancer Res.*, **25**, 270.

NICHOLLS, P. (1961*a*). The action of anions on catalase peroxide compounds. *Biochem. J.*, **81**, 365.

NICHOLLS, P. (1961*b*). The formation and properties of sulphmyoglobin and sulphcatalase. *Biochem. J.*, **81**, 374.

NICHOLLS, P. & SCHONBAUM, G.R. (1963). In: *Catalases, the Enzymes*. (Eds. Boyer *et al.*), New York: Academic Press, Vol. 8, p. 147.

NIE, N.H., HULL, C.H., JENKINS, J.G., STEINBRENNER, K. & BENT, D.H. (1975). *Statistical Package for the Social Sciences, 2nd Ed.*, New York: McGraw-Hill, p. 760.

NISHIMURA, E.T., KOBARA, T.Y., KALTENBACH, J.P. & WARTMAN, W.B. (1962). Immunological evidence of repressed catalase synthesis in livers of tumour bearing mice. *Arch. Biochem. Biophys.*, **97**, 589.

OSHINO, N., CHANCE, B., SIES, H. & BUCHER, TH. (1973). The role of $H_2O_2$ generation in perfused rat liver and the reaction of catalase compound 1 and hydrogen donors. *Arch. Biochem. Biophys.*, **154**, 117.

OSHINO, N., JAMIESON, D. & CHANCE, B. (1975). The properties of hydrogen peroxide production under hyperoxic and hypoxic conditions of perfused rat liver. *Biochem. J.*, **146**, 53.

PRICE, V.E. & GREENFIELD, R.E. (1954). Liver catalase II, catalase fractions from normal and tumour-bearing rats. *J. Biol. Chem.*, **209**, 363.

PURCHASE, H.G. & BURMESTER, B.R. (1972). Leukosis/Sarcoma Group. In: *Diseases of Poultry*. (Eds. Hofstad *et al.*), Ames: Iowa State University Press, p. 502.

SAHA, A., CAMPBELL, D.H. & SCHROEDER, W.A. (1964). Immunochemical studies on liver and erythrocyte catalases from cattle, horse, rabbit and human. *Biochim. Biophys. Acta*, **85**, 38.

THAMM, R., RAPOPORT, S. & NIERADT-HIEBSCH, CH. (1971). Uber die $CO_2$-Bildung aus dem $C_1$-Stoffwechsel von Aszites-Tumorzellen. *Acta Biol. Med. Ger.*, **27**, 459.

WILLIAMS-SMITH, D.L. & MORRISON, P.J. (1975). Electron paramagnetic resonance spectra of catalase in mammalian tissues. *Biochim. Biophys. Acta*, **405**, 253.

WILLIAMS-SMITH, D.L. & PATEL, K. (1975). Induced changes in the electron paramagnetic resonance spectra of mammalian catalases. *Biochim. Biophys. Acta*, **405**, 243.

WOLLENBERGER, A., RISTAU, O. & SCHOFFA, H. (1960). Eine einfache Technik der extrem schnellen Abkuhlung grosserer Gewebstucke, Pflugers Archiv. *Ges. Physiol. Menschen*, **270**, 339.

YAMANAKA, N. & DEAMER, D. (1974). Superoxide Dismutase activity in WI – 38 cell cultures: Effects of age, Trypsinisation and Sv-40 transformation. *Physiol. Chem. Phys.*, **6**, 95.